# Efficacy and Safety of Traditional Chinese Medicine Based on the Method of “Nourishing Kidney and Clearing Heat” as Adjuvant in the Treatment of Diabetes Mellitus Patients with Periodontitis: A Systematic Review and Meta-Analysis

**DOI:** 10.1155/2022/3853303

**Published:** 2022-08-27

**Authors:** Wenqin Jin, Lingfeng Li, Huangping Ai, Zhao Jin, Yuling Zuo

**Affiliations:** ^1^Hospital of Chengdu University of Traditional Chinese Medicine, No. 39 Shi-er-qiao Road, Chengdu, Sichuan 610072, China; ^2^College of Basic Medicine, Chengdu University of Traditional Chinese Medicine, Chengdu, Sichuan, China

## Abstract

**Objective:**

The aim of this systematic review and meta-analysis was to assess the efficacy and safety of traditional Chinese medicine based on the method of “nourishing kidney and clearing heat” as an adjuvant in the treatment of diabetes mellitus patients with periodontitis.

**Methods:**

An electronic literature search was conducted in the China National Knowledge Infrastructure (CNKI), Wanfang Data, Chinese Scientific Journals Database (VIP), Chinese Biomedical Literature Database (CBM), PubMed, EMBASE, Web of Science, and Cochrane Library databases for articles published until October 2021. The primary outcomes were probing pocket depth (PPD), clinical attachment loss (CAL), plaque index (PLI), and sulcular bleeding index (SBI), while the secondary outcomes were tooth mobility (TM), glycosylated hemoglobin (HbA1c), fasting blood glucose (FBG), total effective rate, and adverse effects.

**Results:**

Eleven randomized controlled trials (RCT) were included in the meta-analysis. The pooled results showed PPD (WMD = 1.07, 95%CI: (0.82, 1.33), *P* < 0.00001, *I*^2^ = 89%), CAL (WMD = 0.78, 95%CI: (0.62, 0.93), *P* < 0.00001, *I*^2^ = 58%), PLI (WMD = 0.44, 95%CI: (0.09, 0.79), *P*=0.01, I^2^ = 97%), SBI (WMD = 0.87, 95%CI: (0.79, 0.95), *P* < 0.00001, *I*^2^ = 37%), TM (WMD = 0.26, 95%CI: (0.21, 0.30), *P* < 0.00001, *I*^2^ = 31%), HbA1c (WMD = 0.48, 95%CI: (0.28, 0.67), *P* < 0.00001, I^2^ = 26%), FBG (WMD = 1.34, 95%CI: (0.96, 1.72), *P* < 0.00001, *I*^2^ = 52%), total effective rate (RR = 1.24, 95%CI: (1.14, 1.34), *P* < 0.00001, *I*^2^ = 0%), and adverse effects (RR = 0.78, 95%CI: (0.20, 3.03), *P*=0.72, *I*^2^ = 0%) in the traditional Chinese medicine based on the method of “nourishing kidney and clearing heat” + routine western medicine treatment (periodontal basic treatment, PBT, with or without antibiotic) group were significantly improved compared to control group, but no significant difference was observed for PLI at 2–3 months and 6 months.

**Conclusions:**

This review supports traditional Chinese medicine based on the method of “nourishing kidney and clearing heat” as an adjuvant to routine western medicine treatment in the management of diabetes mellitus patients with periodontitis. Within the limits of the evidence, the well-designed, long-term efficacy, and high-quality multicenter RCTs need to be further confirmed.

## 1. Introduction

Periodontitis is a chronic infectious disease characterized by periodontal tissue destruction and periodontal bone resorption. Epidemiological studies show that about two-third of the world's population suffers from periodontal disease, which is the main cause of adult tooth loss [1] and seriously affects human oral health and quality of life. Diabetes mellitus is a group of metabolic disorders of sugar, fat, and protein caused by insulin resistance or insufficient insulin secretion, and hyperglycemia is its main clinical manifestation [2]. In recent years, researchers have paid more and more attention to the close relationship between periodontitis and diabetes, and other systemic diseases. At present, research shows that periodontal disease is the sixth major complication of diabetes; one-third of diabetic patients suffer from severe periodontal disease, and the possibility of severe periodontitis in diabetic patients is three times than that in nondiabetic patients [3, 4], and both are risk factors for each other [5]. This means that hyperglycemia in diabetes will increase the risk of periodontitis [6–8], a persistent periodontal infection can also increase the difficulty of blood sugar control [9].

At present, western medicine mainly treats diabetic periodontitis patients by controlling blood sugar, removing local pathogenic factors through periodontal basic treatment (PBT, mainly includes supragingival scaling and subgingival scaling), and at the same time, using antibacterial drugs locally or systemically to relieve inflammation of periodontal tissues, and immune regulation treatment[10]. However, mechanical treatment alone cannot remove pathogenic bacteria invading periodontal tissues. Although systemic use of antibiotics and immunosuppressants can control infection, the drug concentration reaching the periodontal pocket is low. Long-term use of antibiotics can produce drug-resistant strains, induce secondary infection, cause gastrointestinal damage, flora imbalance, and other side effects [11]. Traditional Chinese medicine emphasizes the whole differentiation of syndrome, and pays attention to adjusting the whole body state to improve the body's own life level and physical condition, so as to promote local curative effect. Its theory holds that fever due to kidney yin deficiency is the key pathogenesis of diabetic periodontitis, and the application of Chinese medicine based on the method of “nourishing kidney and clearing heat” combined with a periodontal foundation in treating diabetic periodontitis patients has proved that it has obvious advantages in periodontal probe depth (PPD), clinical attachment loss (CAL), sulcular bleeding index (SBI), plaque index (PLI), etc. However, there is no systematic evaluation and meta-analysis on the treatment of diabetes mellitus patients with periodontitis with the help of traditional Chinese medicine at present. Therefore, the objective of this systematic review was to analyze the efficacy and safety of traditional Chinese medicine based on the method of “nourishing kidney and clearing heat” as an adjuvant in the treatment of diabetes mellitus patients with periodontitis.

## 2. Methods

### 2.1. Focus Question and Registration

In order to perform this systematic review, the following question was elaborated: “In diabetes mellitus patients with periodontitis, do traditional Chinese medicine based on the method of ‘nourishing kidney and clearing heat' have an additional clinically meaningful effect when used as an adjuvant to conventional therapy?”.

This meta-analysis was conducted using Review Manager following the Cochrane Handbook for Systematic Reviews of Interventions (version 5.4.1) and the Preferred Reporting Items for Systematic Reviews and Meta-Analyses guidelines. The protocol of this review was registered in INPLASY (INPLASY2021100007).

### 2.2. Search Strategy

Electronic and manual literature searches were conducted by two independent reviewers(WQJ and LFL) using the following databases: China National Knowledge Infrastructure (CNKI), Wanfang Data, Chinese Scientific Journals Database (VIP), Chinese Biomedical Literature Database (CBM), PubMed, EMBASE, Web of science, and Cochrane Library. The articles were searched from database inception until October 2021. There was no restriction to language, systemic conditions of the participants, and publication year. We imposed no minimum follow-up period restriction. The keywords and MeSH terms are as follows: periodontitis, periodontitides, pericementitis, pericementitides, diabetes mellitus, traditional Chinese medicine, tonifying kidney, nourishing yin, clearing heat, nourishing kidney, tonifying kidney, yin deficiency, kidney deficiency, and randomized controlled trial. Any inconsistency was solved by a third reviewer (HPA). The corresponding authors of the included studies might be contacted if additional information was required. The references of retrieved articles were hand-searched to obtain additional eligible articles.

### 2.3. Eligibility Criteria

Two investigators independently screened articles and assessed their eligibility. The included studies met the following inclusion criteria: (1) randomized controlled trials (RCT); (2) adult patients (age > 18) diagnosed with diabetes mellitus with periodontitis; (3) the studies aimed to compare Chinese medicine (based on the method of “nourishing kidney and clearing heat)” + conventional western medicine treatment (periodontal basic treatment(PBT), with or without antibiotic) with conventional western medical treatment alone, no limitations on dosages and course of treatment were set; (4) the trials reported the primary clinical outcome, such as probing pocket depth (PPD) and/or clinical attachment loss (CAL) and/or plaque index (PLI) and/or sulcular bleeding index (SBI). Animal studies, studies with no standard for group comparison, clinical cases, case series, editor letters, abstracts, reviews, and opinion articles were not considered for the analyses and the disagreement was resolved by consulting another reviewer.

### 2.4. Data Extraction

The entire data extraction process of the studies was performed independently by two reviewers (WQJ and LFL). For this, a spreadsheet in Excel was developed specifically for this study, containing variables such as authors, date of publication, country, study design, sample size, average age, gender, intervention measures, follow-up time, and outcome measures. Any conflict was resolved by a third author (HPA). All data were cross-checked and transferred to RevMan software (V.5.4.1).

When the differences (Δ) between baseline-end visits were not reported, they were calculated according to the formula:

ΔVary = Var2−Var1 (Var1 and Var2−mean values before and after treatment).

The variance was estimated with the formula: SVar^2^ = SVar1^2^−SVar2^2^−(2^*∗*^r^*∗*^SVar1^*∗*^SVar2), (SVar12 and SVar22−variances of the mean baseline and end values) [12] (a correlation *r* of 0.5 was assumed) [13].

### 2.5. Outcomes

The primary outcomes preferably evaluated in this systematic review include probing pocket depth (PPD), clinical attachment loss (CAL),plaque index (PLI), and sulcular bleeding index (SBI). The secondary outcomes include tooth mobility (TM), glycosylated hemoglobin (HbA1c), fasting blood glucose (FBG), total effective rate, and adverse effects.

### 2.6. Risk of Bias Assessment

The risk of bias in all included studies was assessed by the Cochrane Handbook for Systematic Reviews which divided them into a low, high, or unclear risk of bias based on the following seven points: (1) random sequence generation; (2) allocation concealment; (3) blinding of participants and personnel; (4) blinding of outcome assessors; (5) incomplete outcome data; (6) selective reporting; and (7) other potential risks of bias. Any divergence was discussed by the third reviewer investigator (HPA).

### 2.7. Meta-Analyses

Meta-analyses were conducted by using the Review Manager 5.4.1 software (The Cochrane Collaboration, NCC, CPH, Denmark) and the results were estimated as the mean difference (MD) with a 95% confidence interval (CI) for continuous variables, and the heterogeneity was assessed by the chi-square and *I*^2^ measurement. In addition, when the result was reported as low heterogeneity (*I*^2^ < 50%), the fixed-effects model was conducted, and when it was estimated as moderate (50% < *I*^2^ < 75%) or high (*I*^2^ > 75%) heterogeneity, the random-effects model was performed. Publication bias assessed by funnel plots at least included 10 trials . Subgroup analyses were performed to identify the potential source of high heterogeneity. Sensitivity analysis was performed to evaluate the stability of the results.

## 3. Result

### 3.1. Study Selection

**Figure 1:**
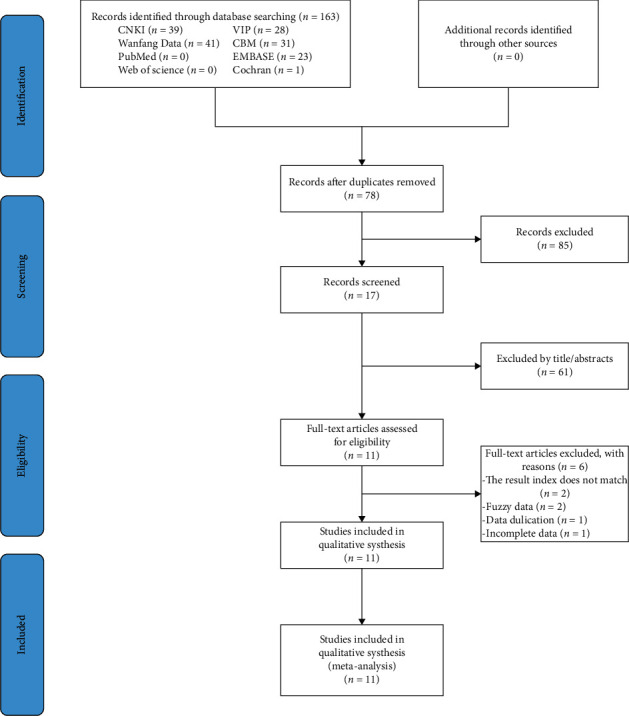
PRISMA flow chart for study selection process.

A total of 163 studies were identified based on the search strategy. After the removal of the duplicates (*n* = 85), an initial screening of titles and abstracts was performed, and 61 articles were excluded as irrelevant to the PICO question. 17 studies were analyzed in full text, and 6 of them were excluded. Eventually, the 11 articles that were remaining were included and processed for data extraction and meta-analysis. The systematic process of the study selection is summarized in the PRISMA flow chart provided in Figure 1.

### 3.2. Characteristics of the Included Studies

The characteristics of the included studies are summarized in Table 1. All selected studies were RCT, and all of them were conducted in China. A total of 1,084 patients were diabetes mellitus patients with periodontal disease, 544 in the intervention group, and 537 in the control group, one of the studies reported shedding [14], including 2 cases in the experimental group and 1 case in the control group. Six studies [15–20] were treated with herbal decoction, and four studies [14, 21–23] were cured with Chinese patent medicine, and in another study [15], fried-free granules were used for treatment. In all of the studies, participants in the test group received traditional Chinese medicine based on the method of “nourishing kidney and clearing heat” + conventional western medicine treatment, while the control group used western medicine treatment alone. For the outcome measurements, the PPD was the most used evaluation method, present in 10 studies [14–20, 22–24], followed by CAL, PLI, and SBI; 4 studies [14, 17, 18, 15] reported the TM, 4 trials presented the HbA1c, 3 studies reported the FBG, 4 trials [22, 17, 18, 15] presented the total effective rate, and 2 trials reported adverse effects [21, 18]. The follow-up period ranged from 14 to 180 days. The composition of traditional Chinese medicine based on the method of “nourishing kidney and clearing heat” prescriptions in the included studies is shown in Supplementary Table 1.

### 3.3. Risk of Bias

**Figure 2:**
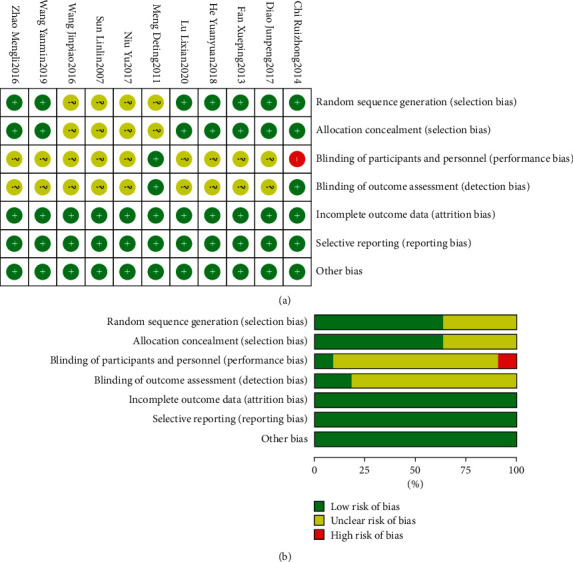
Summary of the risk of bias.

A total of 11 randomized clinical trials evaluated the risk of bias according to COCHRANE criteria, and these data are shown in Figure 2. With regard to the generation of sequences for the randomization of treatments, four studies [16–19] used the random number table method, one [20] was reported to use the method of draw lots, and one [21] used the stratified random method according to age and sex, and one [22] described the use of random parallel grouping method, all of them were classified as low risk of bias. Seven studies [16, 18–23] demonstrated a low risk of bias regarding allocation concealment and five was considered unclear. Regarding the blinding of participant and evaluator, only one study [24] reported the use of the double-blind method, both were considered low risk of bias, and one [20] described the single-blind method (blind evaluator), thus, performance bias was considered high risk, and detection bias was considered low risk, since no blind method was mentioned in the remaining tests, they are demonstrated as unclear bias. All included studies presented a low risk of bias in relation to incomplete outcome data, selective reporting, and other biases, indicating that this information was adequately provided.

### 3.4. Meta-Analysis

The data from the included trials were clubbed together and a meta-analysis was carried out for calculating the mean difference between the intervention and control group for reduction of PPD, PLI, SBI, and gain in CAL, TM, HbA1c, FBG, total effective rate, and adverse effects at all follow-up.

#### 3.4.1. Meta-Analysis for Probing Pocket Depth (PPD)

**Figure 3:**
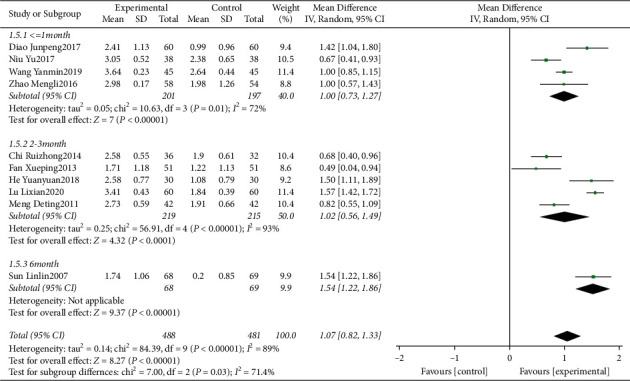
Forest plot for PPD between the experimental and control groups.

Ten clinical trials [14–16, 18–24] evaluating the influence of traditional Chinese medicine based on the method of “nourishing kidney and clearing heat” plus conventional western medicine in the mean of PPD were included. We identified substantial statistical heterogeneity across the included studies (*I*^2^ = 89%). The pooled data of meta-analysis demonstrated that the PPD was significantly reduced in the intervention group (WMD = 1.07, 95%CI: (0.82, 1.33), *P* < 0.00001, *I*^2^ = 89%). Sensitivity analysis showed that the removal of any studies, respectively, from the current analysis did not change the results. Subgroup analyses were performed based on different follow-up times. The pooled data of meta-analysis demonstrated that compared with the control groups, the PPD reduction at ≤1 month (WMD = 1.00, 95%CI: (0.73,1.27), *P* < 0.00001, *I*^2^ = 72%), 2–3 months (WMD = 1.02, 95%CI: (0.56, 1.49), *P* < 0.00001, *I*^2^ = 93%), 6 months (WMD = 1.54, 95%CI: (1.22, 1.86), *P* < 0.00001) (Figure 3) were significantly improved in the intervention groups.

#### 3.4.2. Meta-Analysis for Clinical Attachment Loss (CAL)

**Figure 4:**
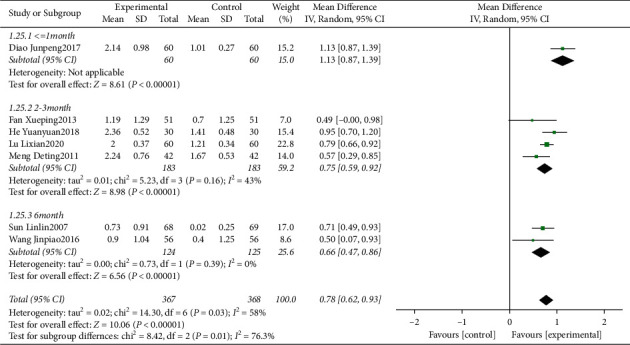
Forest plot for CAL between the experimental and control groups.

Eight clinical trials [14, 17, 19–24] evaluating the influence of traditional Chinese medicine based on the method of “nourishing kidney and clearing heat” plus conventional western medicine in the mean of CAL were included. We identified substantial statistical heterogeneity across the included studies (*I*^2^ = 82%). During sensitivity analysis, the heterogeneity ranges from 0% to 71%, and in an attempt to reduce the overall and subgrouped heterogeneity, the study of Chi Ruizhong^2014^ [20] was excluded from the final analysis. The pooled data of meta-analysis demonstrated that the CAL was significantly improved in the intervention group (WMD = 0.78, 95%CI: (0.62, 0.93), *P* < 0.00001, *I*^2^ = 58%). Subgroup analyses were performed based on different follow-up times. The pooled data of meta-analysis demonstrated that compared with the control groups, the gain of CAL at ≤1 month (WMD = 1.13, 95%CI: (0.87,1.39), *P* < 0.00001), 2-3 months (WMD = 0.75, 95%CI: (0.59, 0.92), *P* < 0.0001, *I*^2^ = 43%), 6 months (WMD = 0.66, 95%CI: (0.47, 0.86), *P* < 0.00001, and *I*^2^ = 0) (Figure 4) were significantly improved in the intervention groups.

#### 3.4.3. Meta-Analysis for Plaque Index (PLI)

**Figure 5:**
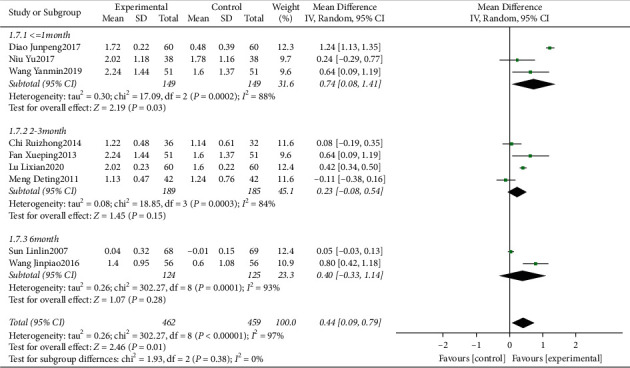
Forest plot for PLI between the experimental and control groups.

Nine clinical trials [14, 15, 17, 18, 20–24] evaluating the influence of traditional Chinese medicine based on the method of “nourishing kidney and clearing heat” plus conventional western medicine in the mean of PLI were included. We identified substantial statistical heterogeneity across the included studies (*I*^2^ = 97%). The pooled data of the meta-analysis demonstrated that the PLI was significantly reduced in the intervention group (WMD = 0.44, 95%CI: (0.09, 0.79), *P*=0.01, *I*^2^ = 97%). Sensitivity analysis showed that the removal of any studies, respectively, from the current analysis did not change the results. Subgroup analyses were performed based on different follow-up times. The pooled data of meta-analysis demonstrated that compared with the control groups, the PLI at ≤1 month (WMD = 0.74, 95%CI: (0.08,1.41), *P*=0.03, *I*^2^ = 88%) were significantly improved in the intervention groups, while at 2-3 months (WMD = 0.23, 95%CI: (−0.08, 0.54), *P*=0.15, *I*^2^ = 84%), 6 months (WMD = 0.40, 95%CI: (−0.33, 1.14), *P*=0.28, and *I*^2^ = 93%) (Figure 5), the difference was not significant.

#### 3.4.4. Meta-Analysis for Sulcular Bleeding Index (SBI)

**Figure 6:**
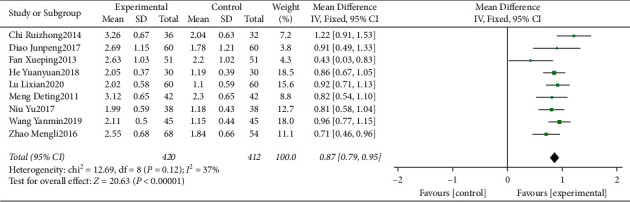
Forest plot for SBI between the experimental and control groups.

Nine clinical trials [15, 16, 18–24] evaluating the influence of traditional Chinese medicine based on the method of “nourishing kidney and clearing heat” plus conventional western medicine in the mean of SBI were included. The heterogeneity was identified as “low” (*I*^2^ = 37%). The pooled data of meta-analysis demonstrated that the SBI was significantly improved in the intervention group (WMD = 0.87, 95%CI: (0.79, 0.95), *P* < 0.00001, *I*^2^ = 37%) (Figure 6).

#### 3.4.5. Meta-Analysis for TM

**Figure 7:**
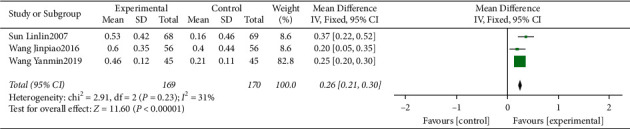
Forest plot for TM between the experimental and control groups.

Four clinical trials [14, 15, 17, 18] evaluating the influence of traditional Chinese medicine based on the method of “nourishing kidney and clearing heat” plus conventional western medicine in the mean of TM were included. We identified substantial statistical heterogeneity across the included studies (*I*^2^ = 86%). During sensitivity analysis, the heterogeneity ranges from 0% to 71%, and in an attempt to reduce the heterogeneity, the study of Niu and Liu^2017^ [15] was excluded from the final analysis. The pooled data of meta-analysis demonstrated that the TM were significantly improved in the intervention group (WMD = 0.26, 95%CI (0.21, 0.30), *P* < 0.00001, *I*^2^ = 31%) (Figure 7).

#### 3.4.6. Meta-Analysis for HbA1c

**Figure 8:**
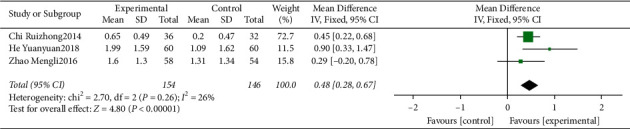
Forest plot for HbA1c between the experimental and control groups.

Four clinical trials [16, 19, 20, 22] evaluating the influence of traditional Chinese medicine based on the method of “nourishing kidney and clearing heat” plus conventional western medicine in the mean of HbA1c were included. We identified substantial statistical heterogeneity across the included studies (*I*^2^ = 95%). During sensitivity analysis, the heterogeneity ranges from 0% to 71%, and in an attempt to reduce the heterogeneity, the study of Lu et al. ^2020^ [22] was excluded from the final analysis. The pooled data of meta-analysis demonstrated that the HbA1c was significantly improved in the intervention group (WMD = 0.48, 95%CI: (0.28, 0.67), *P* < 0.00001, *I*^2^ = 26%) (Figure 8).

#### 3.4.7. Meta-Analysis for FBG

**Figure 9:**
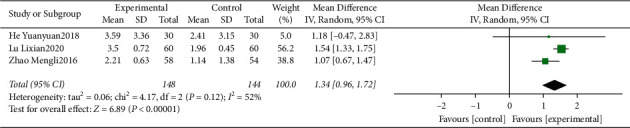
Forest plot for FBG between the experimental and control groups.

Three studies [16, 19, 22] reported a total effective rate. The heterogeneity was identified as “moderate” (*I*^2^ = 52%). The pooled data of meta-analysis demonstrated that the FBG were significantly improved in the intervention group (WMD = 1.34, 95%CI: (0.96, 1.72), *P* < 0.00001, *I*^2^ = 52%) (Figure 9).

#### 3.4.8. Meta-Analysis for Total Effective Rate

**Figure 10:**
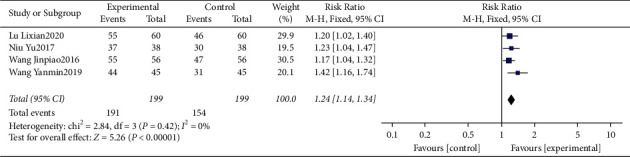
Forest plot for total effective rate between the experimental and control groups.

Four studies [15, 17, 18, 22] reported the total effective rate. The heterogeneity was identified “low” (*I*^2^ = 0%). The data of meta-analysis showed that the interventional group had a significantly higher total effective rate than that control group (RR = 1.24, 95%CI: (1.14, 1.34), *P* < 0.00001, *I*^2^ = 0%) (Figure 10).

#### 3.4.9. Meta-Analysis for Adverse Effects

**Figure 11:**
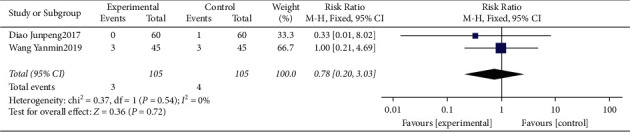
Forest plot for adverse effects between the experimental and control groups.

Two studies [18, 21] reported adverse effects, including nausea, vomiting, diarrhea, and constipation. The heterogeneity was identified as “low” (*I*^2^ = 0%). The data of meta-analysis showed that the adverse effects of the interventional group were significantly lower compared with the control group (RR = 0.78, 95%CI: (0.20, 3.03), *P*=0.72, *I*^2^ = 0%) (Figure 11).

### 3.5. Publication Bias

**Figure 12:**
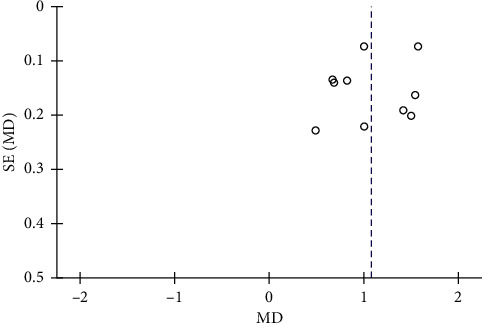
Funnel plots for PPD publication bias.

Funnel plot was used to measure the PPD publication bias, which included more than 10 studies. Also, the figure was in an asymmetric distribution, indicating that publication bias might exist (Figure 12).

## 4. Discussion

Traditional Chinese medicine holds that kidney is innate, and the normal balance of qi, blood, and yin and yang of the whole body depends entirely on the essence of kidney and the sufficiency of yuan, yin, and yang. Diabetes belongs to the category of diabetes in traditional Chinese medicine, which can be divided into upper, middle, and lower consumptions according to its main disease position, among which upper consumptions treat lung, middle consumptions treat stomach, and lower consumptions treat kidney. However, the clinical manifestations of diabetic patients are long-term polydipsia, polyphagia, and diuresis, and the body is in a state of long-term wear and tear. It can lead to the deficiency of primary yin deficiency that cannot subdue the yang, which leads to the hyperactivity of fire. Therefore, in the whole process of the occurrence and development of diabetes, attention should be paid to tonifying the kidney and applying small doses of drugs to clear away the deficiency heat according to the condition of the body. On the basis of nourishing the kidney and subduing the yang, the hyperactivity of fire should be cleared up, so as to take care of the innate essence. At the same time, according to the theory of traditional Chinese medicine, the kidney controls bone marrow, and the teeth are the bones. Therefore, the loss of kidney element in the state of chronic diabetes can directly affect the state of mouth and teeth, and the virtual fire generated by it can also directly lead to periodontal inflammation through the meridian. Therefore, traditional Chinese medicine believes that the treatment of diabetes mellitus with periodontitis should focus on nourishing kidney and clearing heat, or with the help of activating blood circulation, and improve the body's own life level and physical condition by adjusting the whole body state, thereby improving the local state of periodontal, and long-term clinical practice had also confirmed its curative effect.

This systematic review was included in eleven clinical trials studies, and all of them indicated the beneficial effects of traditional Chinese medicine based on the method of “nourishing kidney and clearing heat” as an adjuvant in the treatment of diabetes mellitus patients with periodontitis. Our meta-analyses showed improvement in the parameters of probing pocket depth (PPD), clinical attachment loss (CAL), plaque index (PLI), sulcular bleeding index (SBI), tooth mobility (TM), glycosylated hemoglobin(HbA1c), and fasting blood glucose (FBG). However, since the significant heterogeneity of PPD, CAL, and PLI, we subgrouped in their periodontal analysis based on the follow-up until 1 month, two to three months, and six months in order to compare the periodontal condition before and after the therapies studied, and considering the problems regarding methodological quality, three studies [15, 20, 22] were excluded from the meta-analysis for CAL.

The results of the meta-analyses revealed that using traditional Chinese medicine based on the method of “nourishing kidney and clearing heat” as adjuvants to diabetes mellitus patients with periodontitis improved the following clinical parameters: PPD, CAL, PLI, SBI, TM, HbA1c, and FBG indicating *P* values for the test of significance of the total overall estimate of <0.00001, <0.00001, 0.01, <0.00001, *P* < 0.00001, *P* < 0.00001, and <0.00001, but it should still be noted that the *P* values of PLI at 2-3 months and 6 months were 0.15 and 0.28, that means there is no statistical difference for PLI when the follow-up was 2-3 months or 6 months. We speculated that this may be because Chinese medicine is a systemic medication, and its action point is more inclined to systemic conditioning. Of course, we expect more relevant research to be carried out, so that we can get enough data to verify this.

Despite the evidence of the benefits of traditional Chinese medicine based on the method of “nourishing kidney and clearing heat” as adjuvants in the treatment of diabetes mellitus, patients with periodontitis were definite. But there are still some substantial limitations, such as (1) significant heterogeneity for PPD, CAL, PLI, and TM, the source of the high heterogeneity maybe from different degrees of periodontitis and diabetes mellitus; follow-up times; and although all the traditional Chinese medicines prescription established for nourishing kidney and clearing heat, their compositions are different and their curative effects should be different, and in routine western medicine treatment, the operation of Scaling and Root Planing, the use of antibiotics, and the different dosage or types of antibiotics, etc., all of them can contribute to the heterogeneity. (2) The number of studies included is small and the quality is moderate. (3) Although we have no restriction with language, all included studies publication regions were in China. (4) Studies with negative results were rarely published, as it would cause publication bias.

## 5. Conclusion

In general, this systematic review indicates that traditional Chinese medicine based on the method of “nourishing kidney and clearing heat” are proper adjuvants to the treatment of diabetes mellitus patients with periodontitis and can achieve periodontal local improvement by adjusting the whole body state. This suggests the need for well-designed, long-term, and randomized controlled clinical trials that traditional Chinese medicine as adjuncts to routine western medicine treatment in the treatment of diabetes mellitus patients with periodontitis.

## Figures and Tables

**Table 1:** Basic characteristics of the included study.

Author (year)	Sample size Expt./Ctrl	Average age (y) Expt./Ctrl	Gender male/female		Diagnostics	Intervention measures		Follow-up	Outcomes	
			Expt.	Ctrl		Experiment	Control		Primary	Secondary
Chi (2014)	36/32	51.6/52.1	19/17	17/15	*A* + *B*	Bushen Jianpi Huoxue decoction + PBT + BSC	PBT + BSC	3 m	➀➁➂➃	➅
Diao (2017)	60/60	58.1/57.6	28/32	26/34	*A* + *B*	Bushen decoction + PBT + BSC	PBT + BSC + antibiotic	14 d	➀➁➂➃	➈
Lu (2020)	60/60	61.5/62.8	35/25	33/27	*A* + *B*	Ganluyin decoction + PBT + BSC + AT	PBT + BSC + antibiotic	2 m	➀➁➂➃	➅➆➇
Wang (2016)	56/56	50.4/50.4	58/54		*A* + *B*	Liuwei Dihuang Pills + PBT + BSC	PBT + BSC	6 m	➁➂	➄➇
Sun (2007)	70/70	—	60/80		*A* + *B*	Liuwei Dihuang Pills + PBT + BSC	PBT + BSC	6 m	➀➁➂	➄
Meng (2011)	42/42	56.43/56.43	38/46		*A* + *B*	Qingre Ziyin Huoxue decoction + PBT + BSC	PBT + BSC	2 m	➀➁➂➃	—
Zhao (2016)	58/54	51.56/51.24	28/30	28/26	*A* + *B*	Shuanghua Boheyin decoction + PBT + BSC	PBT + BSC	1 m	➀➃	➅➆
Niu (2017)	38/38	63.8/63.8	45/31		*A* + *B*	Zhibai Dihuang pill + PBT + BSC	PBT + BSC + antibiotic	28 d	➀➂➃	➄➇
Wang (2019)	45/45	54.71/54.45	16/29	16/29	*A* + *B*	Zhibai Dihuang pill + PBT + BSC	PBT + BSC + antibiotic	4 w	➀➂➃	➄➇➈
He (2018)	30/30	53.76/53.72	17/13	16/14	*A* + *B*	Zhibai Dihuang pill + PBT + BSC	PBT + BSC + antibiotic	8 w	➀➁➃	➅➆
Fan (2013)	51/51	54.7/54.2	33/18	29/22	*A* + *B*	Zini Yangyin Qingre Huoxue decoction + PBT + BSC	PBT + BSC + antibiotic	8 w	➀➁➂➃	—

Expt: experimental group; Ctrl: control group; (A): diagnostic criteria for diabetes: It meets the WHO diagnostic criteria for diabetes in 1999, and its types are not limited; (B): diagnostic criteria for periodontitis: it conforms to the diagnostic criteria of chronic periodontitis in the 1999 American Periodontal Classification Standard, and the degree is unlimited; PBT: periodontal basic treatment; BSC: blood sugar control; ➀PD/PPD: probing pocket depth; ➁PLI: plaque index; ➂AL/CAL: clinical attachment loss; ➃SBI: sulcular bleeding index; ➄TM: tooth mobility; ➅HbA1c: glycosylated hemoglobin; ➆FBG: fasting blood glucose; ➇total effective rate; ➈adverse effects.

## Supplementary Materials

Supplementary Materials Supplementary Table 1: composition of TCM prescriptions in the included studies.
