# Supplementary material for: Efficacy and Safety of Traditional Chinese Medicine Based on the Method of “Nourishing Kidney and Clearing Heat” as Adjuvant in the Treatment of Diabetes Mellitus Patients with Periodontitis: A Systematic Review and Meta-Analysis
Source: Evid Based Complement Alternat Med. 2022 Aug 27;2022:3853303. doi: 10.1155/2022/3853303 (PMC9440795; doi:10.1155/2022/3853303)
Supplement: Supplementary Materials — Supplementary Table 1: composition of TCM prescriptions in the included studies. [file 3853303.f1.docx]

| Author  Year | Prescription name | medications | form |
| --- | --- | --- | --- |
| Chi^2014^ | Bushen Jianpi Huoxue Decoction | Radix Rehmanniae Preparata 15g, Corni Fructus 10g, Rhizoma Dioscoreae 15g, Cortex Moutan 10g, Alismatis Rhizoma 15g, Poria cocos 15g, Radix Astragali 20g, Achyranthis Radix 15g, Radix Salviae Miltiorrhizae 15g, and Rhizoma Coptidis 15g. | Water decoction |
| Diao^2017^ | Bushen Decoction | Gypsum 10g, scutellaria 10g, coptis 10g, radix rehmanniae 10g, radix rehmanniae preparata 15g, cortex moutan 15g, corni fructus 10g, poria 10g, alismatis Rhizoma 10g, fructus lycii 10g, Rhizoma drynariae 15g, and radix astragali 20g. | Water decoction |
| Lu^2020^ | Ganluyin Decoction | Radix Rehmanniae, Radix Rehmanniae Preparata and Scutellariae each 15 g, Radix Ophiopogonis, Radix Asparagi and Herba Dendrobii each 10g, Fructus Aurantii, Herba Artemisiae Scopariae and Folium Eriobotryae each 8 g, and Radix Glycyrrhizae Preparata 6 g. | Water decoction |
| Wang^2016^ | Liuwei Dihuang Pills | Radix Rehmanniae Preparata, Fructus Corni, Rhizoma Dioscoreae, Alismatis Rhizoma, Cortex Moutan and Poria. | Chinese patent drug |
| Sun^2007^ | Liuwei Dihuang Pills | Radix Rehmanniae Preparata, Fructus Corni, Rhizoma Dioscoreae, Alismatis Rhizoma, Cortex Moutan and Poria. | Chinese patent drug |
| Meng^2011^ | Qingre Ziyin Huoxue Decoction | Honeysuckle 15g, Glehnia littoralis 15g, Chuanxiong 15g. | Water decoction |
| Zhao^2016^ | Shuanghua Boheyin Decoction | Honeysuckle 20g, mint 15g, Anemarrhena asphodeloides 20g, dahurian angelica root 15g and phellodendron amurense 15g. | Water decoction |
| Niu^2017^ | Zhibai Dihuang Pill | Cortex Phellodendri, Rhizoma Anemarrhenae, Radix Rehmanniae Preparata, Fructus Corni, Rhizoma Dioscoreae, Alismatis Rhizoma, Cortex Moutan and Poria. | Fried-free granules |
| Wang^2019^ | Zhibai Dihuang Pill | Cortex Phellodendri 12g, Rhizoma Anemarrhenae 10g, Radix Achyranthis Bidentatae 12g, Radix Rehmanniae Preparate 12g, Cortex Moutan 10g, Rhizoma Drynariae 30g, Gypsum 30g. | Chinese patent drug |
| He^2018^ | Zhibai Dihuang Pill | Cortex Phellodendri, Rhizoma Anemarrhenae, Radix Rehmanniae Preparata, Fructus Corni, Rhizoma Dioscoreae, Alismatis Rhizoma, Cortex Moutan and Poria. | Chinese patent drug |
| Fan^2013^ | Zini Yangyin Qingre Huoxue Decoction | Radix Rehmanniae Preparata 15g, Corni Fructus 12g, Rhizoma Dioscoreae 12g, Flos Lonicerae 15g, Rhizoma Chuanxiong 15g, Alismatis Rhizoma 12g, Cortex Moutan 12g, Poria cocos 12g. | Water decoction |

Supplementary Table 1 Specific prescription
